# Assessment of solitary pulmonary nodules using dual-layer spectral detector computed tomography

**DOI:** 10.1097/MD.0000000000040014

**Published:** 2024-10-11

**Authors:** Tse-Pang Ko, Yu-Pin Chang, Jyh-Wen Chai

**Affiliations:** a Department of Radiology, Wuri Lin Shin Hospital, Taichung, Taiwan; b Premium Health Examination Center, Tungs’ Taichung MetroHarbor Hospital, Taichung, Taiwan; c Department of Radiology, Taichung Veterans General Hospital, Taiwan.

**Keywords:** dual-energy spectral computed tomography, iodine concentration maps, solitary pulmonary nodules, virtual monochromatic images

## Abstract

We aim to quantitatively investigate the difference between benign and malignant solid pulmonary nodules that appeared on dual-energy spectral computed tomography, and assess the diagnostic accuracy of several parameters derived from computed tomography in differentiating malignant from benign pulmonary nodules. Between September 2021 and December 2022, spectral images of 71 patients (male:female = 44:27, mean age = 71.0 years) confirmed by pathology were retrospectively analyzed in the venous phase. Patients were classified into the malignant group and the benign group. The iodine concentration values of the nodules, normalized iodine concentration of the nodules to the neighboring vessels, virtual monochromatic images of 40 and 80 keV, and slope of the spectral curve were calculated and compared between the benign and malignant groups. Receiver operating characteristic curves and the area under the curve were performed to assess the diagnostic performance of the above parameters. Both virtual monochromatic images and iodine concentration maps prove to be highly useful in differentiating benign and malignant pulmonary nodules. The malignant pulmonary nodules have higher iodine density and slope of the spectral curve than the benign lesions. The combined model of iodine density and curve slope with an optimal cutoff of 0.39 (area under the curve = 0.82) yielded a sensitivity of 95% and a specificity of 63%. Contrast-enhanced dual-energy spectral computed tomography allows promising capability of distinguishing malignant from benign lesions, potential for avoiding unnecessary invasive procedure or surgery.

## 1. Introduction

Primary lung cancer is 1 of the most prevalent type of malignancies and a significant contributor to cancer-related deaths worldwide, accounting for approximately 23% of total cancer mortalities.^[1]^ Treatment options for lung cancer include surgery, chemotherapy, radiotherapy, and target therapy, which are determined based on the stage and operability of the disease. Early diagnosis and timely treatment of lung cancer are widely emphasized globally. However, the lungs also commonly harbor nodules with benign characteristics, such as vascular, infectious, and congenital lesions. The treatment options for these conditions typically involve the use of steroids and antibiotics, while unnecessary pulmonary resections should be avoided. Consequently, accurate differentiation between malignant and benign lesions of lung parenchyma becomes crucial due to distinct therapeutic approaches required.

In imaging studies, primary lung cancer is predominantly observed as solitary pulmonary nodules (SPNs) on computed tomography (CT). Low-dose CT has recently been recommended for lung cancer screening, resulting in the early detection of SPNs. Traditionally, whenever a pulmonary lesion is suspected, the patient is referred for further assessment using conventional multidetector-row computed tomography (MDCT).

With conventional CT malignancy is primarily diagnosed based on the morphology, interfaces, inner densities, and enhancement characteristics of the lesions.^[2]^ However, distinguishing between benign and malignant lesions still remains very challenging due to significant image overlapping and similarity between them.^[3,4]^ Degree of enhancement may provide valuable information regarding the pulmonary lesions. Some studies claims that inflammatory benign nodules has strong enhancement on arterial phase and washout on venous phase due to its supply from the bronchial arteries and active lymphatic drainage. However, dynamic contrast-enhanced CT increased radiation dose to patients.^[5,6]^ A prospective and multicenter study involving 356 pulmonary nodules demonstrated that conventional contrast-enhanced CT has a high sensitivity of up to 98% in detecting both benign and malignant pulmonary nodules, though with relative poor specificity of 58%.^[7]^

Recently, dual-energy spectral computed tomography (DESCT) imaging modes, with dominantly kVp-switching and dual-source techniques, have been introduced. These modes have the capability to generate monochromatic spectral images across a range of energy levels from 40 to 140 keV and quantify iodine concentration (IC). In addition to the mentioned parameters, effective atomic numbers and material decomposition images have been utilized in multiparameter and quantitative analysis. These tools aid in distinguishing between various material compositions.

The latest dual-layer spectral detector CT offers the advantage of eliminating the need to preset a dual-energy protocol for acquiring all spectral datasets, which significantly improves work efficiency. Additionally, its system utilizes anticorrelated noise suppression coupled with an iterative algorithm, leading to reduced image noise and improved overall image quality.^[8–10]^ The utilization of low-energy virtual monochromatic images (VMIs), known for their contrast-enhancing properties, plays a pivotal role in minimizing the required contrast media, particularly advantageous for patients with renal insufficiency.

However, there is limited research exploring the quantitative parameters of DESCT for distinguishing between pulmonary malignant nodules and benign nodules. We aim to quantitatively investigate the difference of benign and malignant solid pulmonary nodules appeared on DESCT, and assess the diagnostic accuracy of several parameters derived from DESCT in differentiating malignant from benign pulmonary nodules.

## 2. Materials and methods

### 2.1. Patients

**Figure 1.**
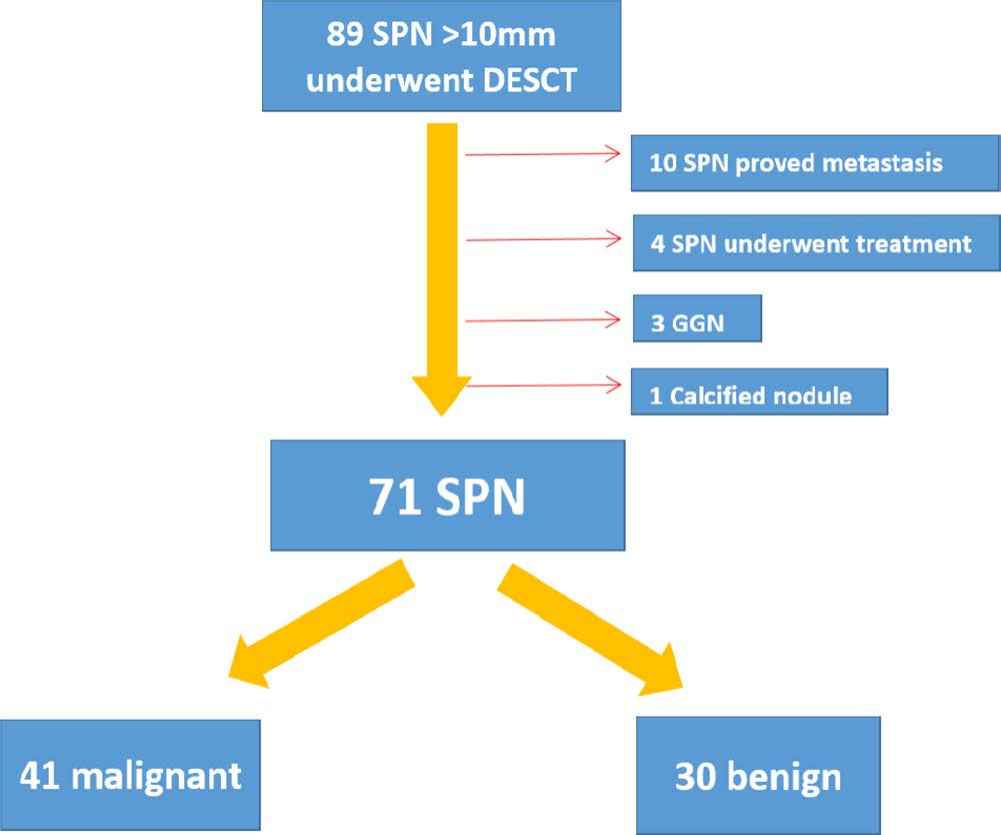
Flow chart of patient selection. DESCT, dual-energy spectral computed tomography; SPN, solitary pulmonary nodule; GGN, ground-glass nodule.

We collected patients who underwent DESCT venous phase enhanced chest scans in our hospital from September 2021 to December 2022 and those with SPNs between 10 mm and 30mm in the lung window were included in our study(n = 89). Eighteen out of 89 patients were excluded for the following reasons: patients who had a primary tumor in another organ or had pathology indicating metastasis (n = 10); patients who received chemoradiotherapy prior to the DESCT scan (n = 4); nodules appearing as pure ground-glass opacity (pGGO) or as subsolid nodules in the lung window (n = 3); the lesion was predominantly composed of calcification (n = 1) (Fig. 1).

As a result, a total of 71 patients (44 men and 27 women) were finally included in our study. The age range of the patients was 19 to 88 years, with a median age of 71 years. Among them, 41 patients were proved to have primary lung cancers according to surgery following the DESCT image study, while 30 patients had benign lesions consisting of inflammatory or congenital abnormalities. All malignant lesions (n = 41) and the majority of benign lesions (n = 27) were pathologically confirmed. Three benign lesions were confirmed through sputum culture, along with clinical diagnosis demonstrated regressive changes after anti-infective therapy and a 3-6 months follow-up. Detailed information regarding patient characteristics and pathological results were summarized in Tables 1 and 2. The retrospective study was approved by ethics committee of our hospital (No. CE23198B).

**Table 1:** Nodules diagnoses and numbers.

	Number (%)
Malignant	41
Small cell lung cancer (SCLC)	3 (7%)
Squamous cell carcinoma (SCC)	8 (20%)
Adenocarcinoma	27 (66%)
Neuroendocrine carcinoma	1 (3%)
Large cell carcinoma	2 (4%)
Benign	30
Chronic inflammation	10 (33.3%)
Fungal infection	6 (20%)
Pulmonary abscess/septic emboli	3 (10%)
Non-tuberculous mycobacterial (NTM)	1 (3.3%)
Pulmonary sequestration	2 (6.6%)
Solitary fibrous tumor	1 (3.3%)
Schwannoma	1 (3.3%)
Bronchogenic cyst	1 (3.3%)
Chondroid hamartoma	2 (6.6%)
Pulmonary pneumocytoma	1 (6.6%)
Intrapulmonary lymph node	2 (6.6%)

**Table 2:** Comparison of patients’ clinical characteristics between groups.

	malignant (n = 41)	benign (n = 30)	*p* value
Gender			
Men	26 (63.4%)	18 (60.0%)	0.086*
Women	15 (36.6%)	12 (40.0%)	
Age(year)	66.95 ± 12.02	52.96 ± 17.59	0.061†
Smoking history			
Yes	15 (36.6%)	5 (25.0%)	0.065*
No	26 (63.4%)	15 (75.0%)	

* Chi-squared test.

† Two-independent-samples Student *t* test

### 2.2. CT examinations

CT examinations were conducted for all patients using a dual-layer spectral detector CT (IQon Spectral CT, Philips Healthcare, Cleveland, OH, USA). The patients were positioned in a supine position, and the scanning range covered the lower thyroid gland to the bilateral adrenal glands, ensuring complete coverage of all lung tissues. To enhance the scan, 100 mL of nonionic iodinated contrast material (Xenetix, 350 mg/mL; Guerbet, Aulnay-sous-Bois, France) was injected into the antecubital vein at a rate of 2.2 mL/s using a power injector. The enhancement scan delay time with GSI mode was set to 100 seconds after the initiation of contrast medium injection, during the venous phase. Patients were trained in breath-holding techniques before the examination to minimize motion artifacts during the scan.

The other CT parameters were as follows: tube voltage: 120 kV; automatic tube current modulation with the Dose Right Index set to 19 (this value was selected to achieve a balance between image quality and radiation dose); rotation speed: 0.33 s/rot; helical pitch: 1.1; detector collimation: 64 × 0.625 mm; image reconstruction matrix: 512 × 512; reconstruction kernel: Sharp (C) for mediastinal window and lung window. All images were reconstructed as Spectral Base Image datasets, with the spectral reconstruction level set to 3. The reconstructed slice thickness was 1 mm with an increment of 0.6 mm. Images reconstructed using the Sharp kernel (C) were evaluated in a mediastinal window with a window width of 450 HU and a level of 60 HU, while those reconstructed using the Sharp kernel (C) were assessed in the lung window with a window width of 1200 HU at a level of −600 HU.

### 2.3. Radiation dose

The volumetric CT dose index (CTDIvol) for each phase during dual-energy CT (DECT) acquisition was a constant value of 9.60 mGy, as our system did not have automatic exposure control available for the DECT acquisition mode. The dose-length product value was recorded as 288.30 ± 48.20 mGy.cm.

### 2.4. Quantitative analysis

The images were analyzed using a proprietary image workstation (IntelliSpace Portal 6.5, Philips Healthcare, Taipei, Taiwan).

VMIs at 40 keV and 80 keV, as well as iodine density maps, were utilized. A region of interest with an area of 50 mm² was manually delineated at the center of the nodules in the relatively homogeneous tissue. The delineation of region of interest was independently performed by a radiologist with 4 years of experience in thoracic imaging.

The following parameters were employed in our study: CT value (HU) from 40 keV and 80 keV reconstructions (CT40keV and CT80keV); the delta of the spectral HU curve (delta HU): CT40keV - CT80keV; the slope of the spectral HU curve (slope HU): |CT40keV-CT80keV|/(80–40); 4. the CT value of the conventional image; the IC; normalized iodine concentration to the aorta (NICa): the IC values of the SPN were normalized to those of the thoracic descending aorta in the same slice; normalized iodine concentration to the pulmonary artery (NICpa): the IC values of the SPN were normalized to the bifurcation of pulmonary trunk; normalized iodine concentration to the pulmonary vein (NICpv): the IC values of the SPN were normalized to nearby left/right superior/inferior pulmonary vein in the neighboring slice.

### 2.5. Dual-energy CT imaging characteristics

**Figure 2.**
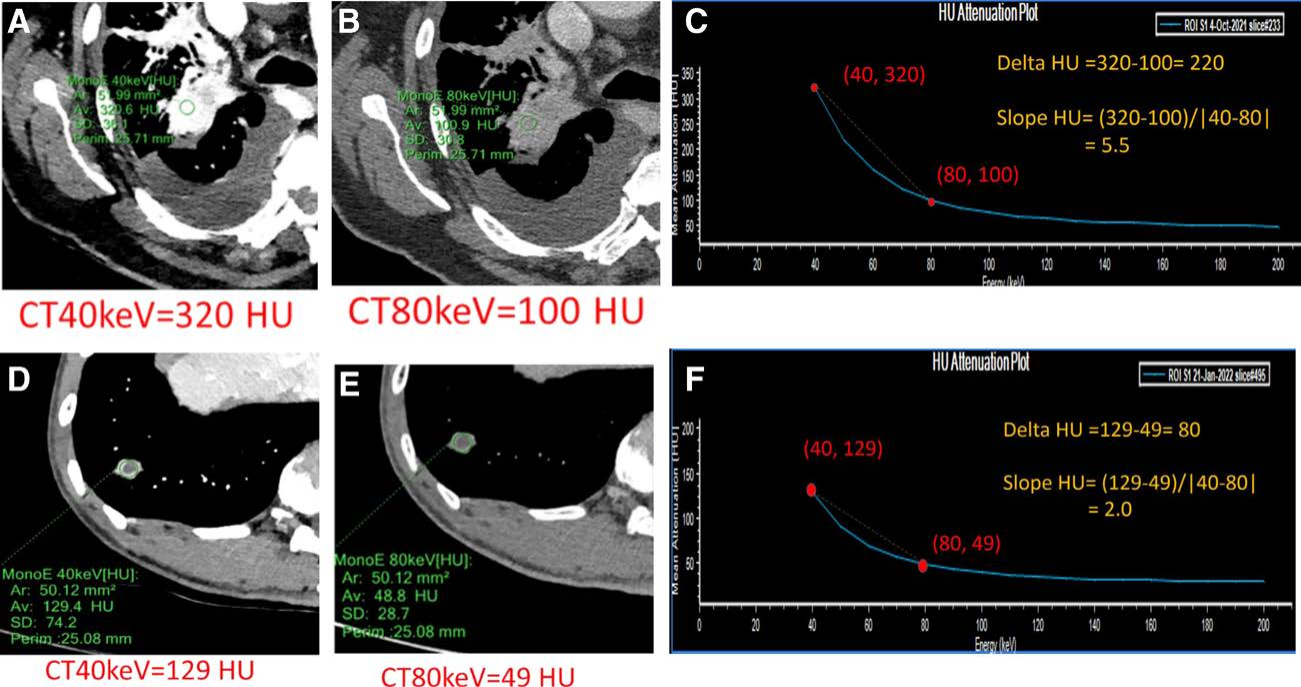
Spectral CT images of a 66-year-old male with adenocarcinoma are shown in panels (A) to (C), while images of a 63-year-old male with an abscess are shown in panels D to F. Panels (A) and (D) display 40 keV images after enhancement, panels (B) and (E) show 80 keV images after enhancement, and panels (C) and (F) depict spectral curves. The CT40keV values are 320 HU for adenocarcinoma and 129 HU for the abscess. The CT80keV values are 100 HU for adenocarcinoma and 49 HU for the abscess. The delta HU values are 220 HU for adenocarcinoma and 80 HU for the abscess. The slope HU values are 5.5 for adenocarcinoma and 2 for the abscess. keV, kilo electron volt; CT40keV and CT80keV, CT value from 40 keV and 80 keV reconstructions; Delta HU, CT40keV-CT80keV; Slope HU, delta HU/(80-40).

**Figure 3.**
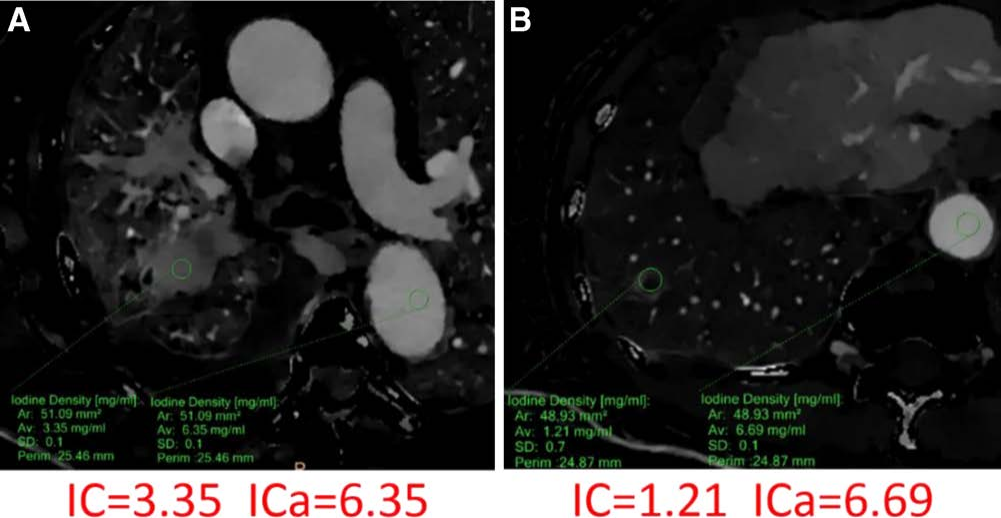
Spectral computed tomography presents iodine-based images of a 66-year-old male with adenocarcinoma (A) and a 63-year-old male with an abscess (B). The IC of the lesion is 3.35 mg/mL, the ICa is 6.35 mg/mL, and the NICa is 0.528 for the adenocarcinoma case. For the abscess case, the IC is 1.21 mg/mL, the ICa is 6.69 mg/mL, and the NICa is 0.18. IC, iodine concentration; ICa, iodine concentration of the aorta; NICa, normalized iodine concentration to the aorta.

Examples of CT number series parameters derived from spectral CT curves in 2 patients with pulmonary malignant lesions and inflammation are presented in Figure 2. Additionally, Figure 3 displays the parameters of IC series in 2 patients with pulmonary malignant lesions and inflammation.

### 2.6. Statistical analysis

In the end, SPSS 22.0.0 (IBM, Armonk, NY, USA) was utilized for all statistical analyses involving CT40keV, CT80keV, delta HU, slope HU, conventional image, IC, and NIC. Continuous variables were presented as means ± standard deviations or medians with interquartile ranges. The Chi-squared test, 2-independent-samples Student *t* test, or Mann–Whitney *U* test were employed to compare the differences in spectral parameters between the benign and malignant groups. Receiver operating characteristic (ROC) curves and the area under the ROC curve (AUC) of different parameters were used to assess their diagnostic ability in differentiating benign and malignant nodules. The Youden index was calculated to determine the parameter thresholds for distinguishing SPNs. Sensitivity, specificity, accuracy, positive predictive value, and negative predictive value were calculated to evaluate the diagnostic performance. Combined model of either 2 of the 3 quantitative parameters (IC, slope HU, and conventional image) were assessed using multivariable regression analyses to figure out the best parameter combination for distinguishing benign from malignant nodules. Statistical significance was defined as *P* ≤ .05.

## 3. Results

**Figure 4.**
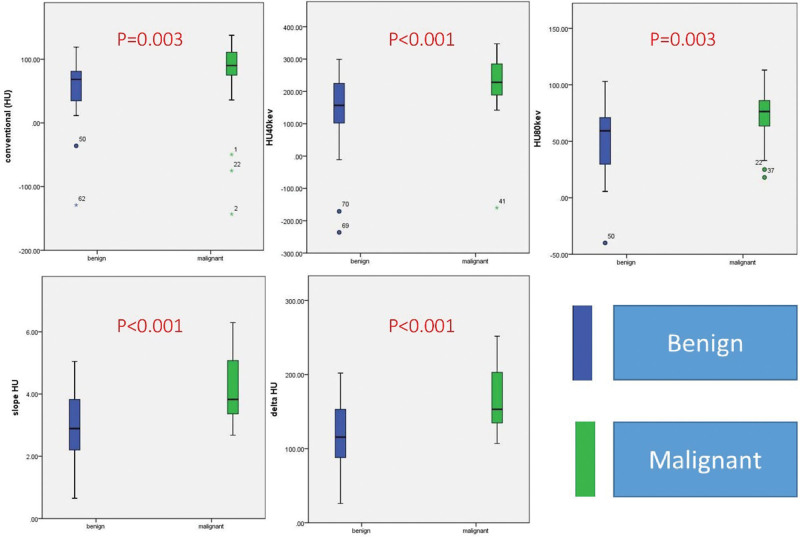
The difference of benign and malignant nodules on evaluating their conventional attenuation, CT40keV, CT80keV, delta HU, and slope HU. The malignant nodules demonstrate higher value on all parameters as compared with benign nodules (all *P* < .05). keV, kilo electron volt; CT40keV and CT80keV, CT value from 40 keV and 80 keV reconstructions; Delta HU, CT40keV-CT80keV; Slope HU, delta HU/ (80-40).

**Figure 5.**
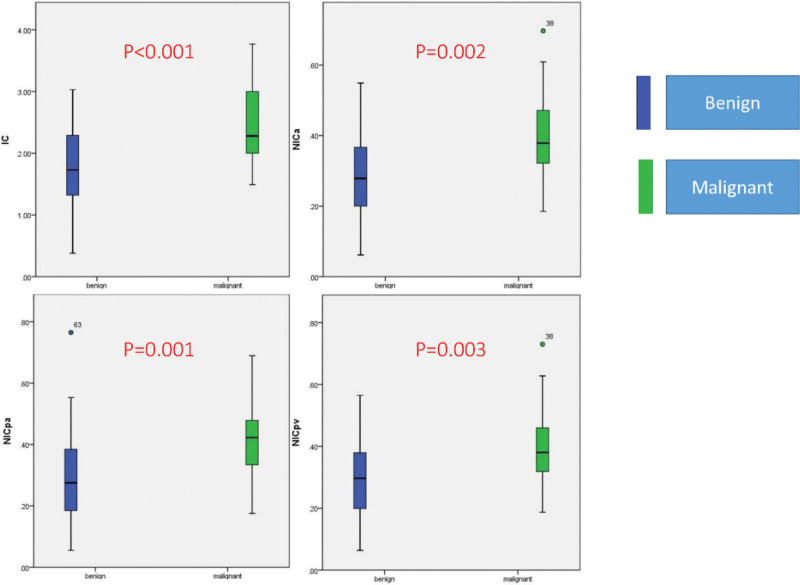
The difference of benign and malignant nodules on evaluating their IC, NICa, NICpa, NICpv. The malignant nodules demonstrate higher value on all parameters as compared with benign nodules (all *P* < .05). IC, iodine concentration; NICa, normalized iodine concentration to the aorta; NICpa, normalized iodine concentration to the pulmonary artery; NICpv, normalized iodine concentration to the pulmonary vein.

Benign and malignant solid pulmonary nodules are compared on the parameters of CT number series (conventional HU, CT40keV, CT80keV, delta HU, slope HU) and IC series(IC, NICa, NICpa, and NICpv). The malignant nodules demonstrate higher value (*P* < .05) on all parameters than the benign nodules, especially on CT40keV, delta HU, slope HU and IC (*P* < .001) (Table 3, Figs 4 and 5).

**Table 3:** Quantitative parameters.

	malignant (n = 41)		benign (n = 30)		*P* value
	mean	95%CI	mean	95%CI	
Conventional image (HU)	90.00	(75.00–111.50)	68.30	(31.70–82.85)	0.003*
CT40kev (HU)	228.00	(188.40–289.00)	157.05	(98.50–226.00)	<0.001*
CT80kev (HU)	76.40	(61.25–89.50)	59.20	(27.78–71.88)	0.003*
Delta HU (HU)	153.00	(133.80–203.00)	115.60	(87.75–154.00)	<0.001*
Slope HU	3.83	(3.35–5.08)	2.89	(2.19–3.85)	<0.001*
IC (mg/mL)	2.28	(2.00–3.02)	1.73	(1.31–2.30)	<0.001*
NICa	0.38	(0.32–0.47)	0.28	(0.20–0.37)	0.002*
NICpa	0.42	(0.32–0.48)	0.28	(0.18–0.39)	0.001*
NICpv	0.38	(0.32–0.46)	0.30	(0.20–0.39)	0.003*

Mann–Whitney *U* test.

CT40keV and CT80keV = CT value from 40 keV and 80 keV reconstructions, Delta HU = CT40keV-CT80keV, IC = iodine concentration, keV = kilo electron volt, NICa = normalized iodine concentration to the aorta, NICpa = normalized iodine concentration to the pulmonary artery, NICpv = normalized iodine concentration to the pulmonary vein, Slope HU = delta HU/ (80-40).

* *P* < 0.01.

**Figure 6.**
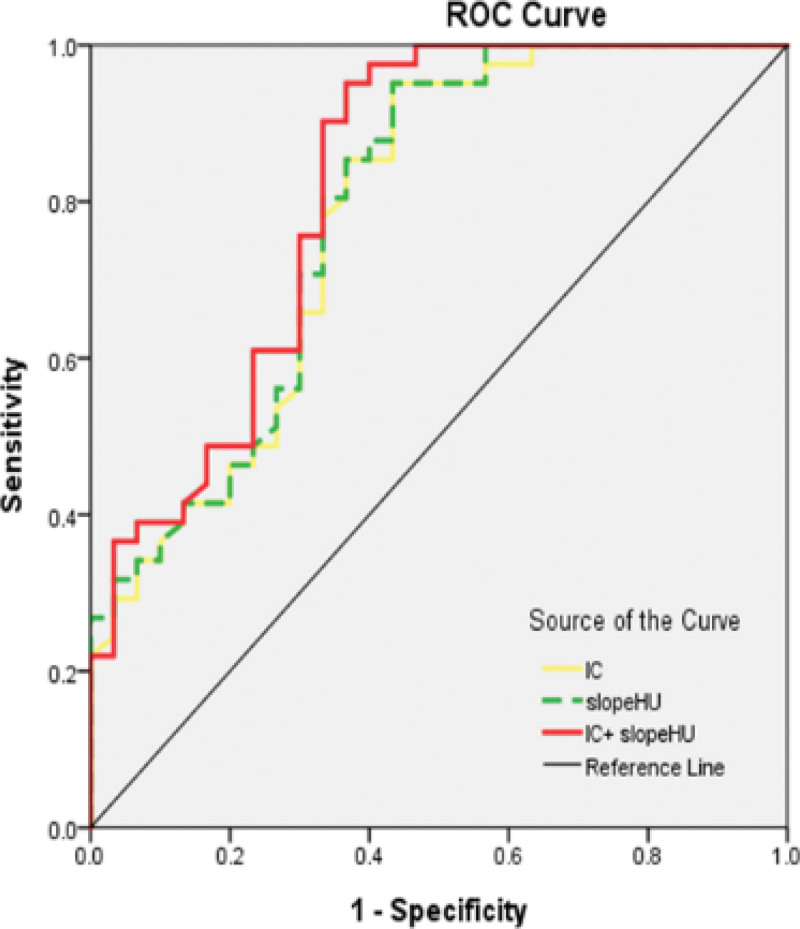
ROC curves for distinguishing benign lesions from malignant lesions using parameters of IC and slope HU (AUC = 0.78 and 0.79). The multivariate combined model of slope HU and IC increases AUC to 0.82. ROC, receiver operating characteristic curves; IC, iodine concentration; keV, kilo electron volt; Slope HU, |CT value of 40 keV reconstruction – CT value of 80 keV reconstruction|/ (80–40). AUC, area under the curve.

The ROC curves of CT number series and IC series for differentiating lung cancers from benign lesions were shown in Table 4. All the ROC curves are above the reference line. The AUC for slope HU and delta HU of lung nodules is 0.79, which is greater than the AUC for IC (AUC = 0.78) and other quantitative parameters. The optimal threshold values of all parameters are calculated by using the ROC curves to optimize both sensitivity and specificity for differentiating benign and malignant lung nodules. When a threshold value of 2.9 is selected for slope HU, a sensitivity of 95.1% and specificity of 56.7% are noted, respectively. In comparison to slope HU alone, a combination of models of slope HU and IC leads to an increase in AUC to 0.82, with sensitivity of 95.10% and specificity of 63.3% (Table 4 and Fig. 6).

**Table 4:** Receiver operating characteristic analyses of dual-energy spectral computed tomography quantitative parameters, and combined model.

Variables	AUC	(95% CI)	*P*	Optimal cutoff	Sensitivity (%)	Specificity (%)
Conventional image (HU)	0.71	(0.59–0.81)	.001	>81.00	63.4%	76.7%
HU40kev (HU)	0.77	(0.66–0.86)	<.001	>179.00	92.7%	60.0%
HU80kev (HU)	0.71	(0.59–0.81)	.001	>71.00	63.4%	76.7%
Delta HU (HU)	0.79	(0.68–0.88)	<.001	>116.00	95.1%	56.7%
Slope HU	0.79	(0.68–0.88)	<.001	>2.90	95.1%	56.7%
IC (mg/mL)	0.78	(0.67–0.87)	<.001	>1.74	95.1%	56.7%
NICa	0.72	(0.60–0.82)	.001	>0.32	75.6%	63.3%
NICpa	0.73	(0.61–0.83)	.001	>0.23	95.1%	46.7%
NICpv	0.70	(0.58–0.81)	.002	>0.24	95.1%	46.7%
IC and conventional image	0.79	(0.67–0.90)	<.001	>0.50	82.9%	66.7%
IC and slope HU	0.82	(0.72–0.92)	<.001	>0.39	95.1%	63.3%
Conventional image and slope HU	0.79	(0.68–0.90)	<.001	>0.42	90.2%	60.0%

AUC = area under the receiver operating characteristic curve, CI = confidence interval, CT40keV and CT80keV = CT value from 40 keV and 80 keV reconstructions, Delta HU = CT40keV-CT80keV, IC = iodine concentration, keV = kilo electron volt, NICa = normalized iodine concentration to the aorta, NICpa = normalized iodine concentration to the pulmonary artery, NICpv = normalized iodine concentration to the pulmonary vein, Slope HU = delta HU/(80-40).

## 4. Discussion

### 4.1. Category of dual energy

Recently, conventional single energy CT is gradually replaced by DECT, which utilizes 2 separate x-ray photon energy spectra to differentiate materials based on their different attenuation properties at different energies. This data could be used to reconstruct various types of images.

There are different acquisition techniques to obtain dual-energy data from various vendors. The 3 most popular approaches include dual-source, dual-energy CT (Siemens Healthcare, Forchheim, Germany); fast kVp switching (GE Healthcare, Milwaukee, WI); and dual-layer detectors (Philips Medical Systems, Cleveland, OH).^[11]^

In our study, we utilized dual-layer DESCT (Philips vendor). The top-layer detector absorbs low-energy x-ray photons, while the high-energy photons pass through the bottom-layer detector and are detected. By analyzing the 2 simultaneous x-ray spectra, dual-energy imaging allows the representation of any material using a basis material pairs, resulting in the formation of IC image and VMIs at different single energy levels. We applied different manifestations in IC and VMIs to distinguish benign lesions from malignancies.

### 4.2. Our study result

The results of this study demonstrate significant differences in the iodine-related parameters of CT40keV, delta HU, slope HU, IC, and NICpa obtained from dual-layer DECT between benign and malignant nodules. For optimal cutoff values, delta HU of 116HU and slope HU of 2.9 exhibit the best diagnostic performance (AUC = 0.79) in our study as single variance parameter, with a sensitivity of 95% and a specificity of 57%. Additionally, iodine density of 1.74 mg/mL (AUC = 0.78) yields a specificity of 95% and a sensitivity of 57%. CT40keV of 179 (AUC = 0.77) yields a specificity of 93% and a sensitivity of 60%. NICpa of 0.23 (AUC = 0.73) yields a specificity of 95% and a sensitivity of 46%. These results suggest that the iodine content after enhancement in nodules can serve as a quantitative parameter to distinguish malignant nodules from benign ones.

It is observed that the groups with VMIs parameters precede to the groups with IC parameters and NIC. Among the VMIs parameter groups, CT40keV shows superiority over CT80keV, and delta/slope HU exhibit superiority over CT40keV/CT80keV. In the IC parameter group, IC shows better performance than NIC. Furthermore, NICpa surpass NICa and NICpv. Almost all the IC and VMIs parameter groups demonstrate superiority over conventional CT number parameters.

### 4.3. Difference with other study

#### 4.3.1. VMIs

In a previous study using fast kVp-switching DECT, CT 70keV was found to be a good parameter for discriminating lesions. This is due to 2 reasons. Firstly, CT values on the 70 keV monochromatic image in kVp-switching dual-energy CT are similar to those on the 120 kVp conventional polychromatic images. Secondly, lower keV monochromatic images have a noise effect that can impact image quality.^[12]^

The strong photoelectric effect of iodine at lower energies and the increased uptake of iodine in malignancy allow us to differentiate malignant lesions from benign lesions.^[13]^ The use of lower keV on monochromatic images makes it easier to recognize malignant lesions. However, lower keV images also have increased image noise, which limits their potential for diagnosing diseases.^[14]^

Fortunately, with spectral dual-layer detectors (Philips), spatially and temporally matched high- and low-energy projection data can be used to suppress noise and improve image quality in monochromatic images with lower keV.^[15]^

In our study, we selected CT40keV as the lower keV parameter and CT80keV as the higher keV parameter. As predicted, CT40keV proved to be more effective in characterizing lesions compared to CT80keV. Similar to the studies conducted by Zegadlo et al and Wen et al, our results showed AUC values for CT with low keV in the range of 0.78-0.89 using different dual-energy CT techniques.^[13,16]^

#### 4.3.2. Slope HU

Each tissue has its characteristic HU curve, providing valuable information about the attenuation characteristics of the X-ray beam as it passes through different tissues, which is closely related to the chemical composition of those tissues. The slope of the spectral curve allows us to assess the attenuation characteristics.^[17]^ Another perspective suggests that the slope disparity between the 2 types of the enhancing pulmonary nodules in low keV images may be attributed to the increased attenuation of iodine at lower energy level.^[18]^ This phenomenon can lead to greater contrast and differentiation between the nodules. Using the spectral curve for disease differentiation has been explored by some researchers.^[17]^ A recent study highlighted a notable discrepancy in the spectral curve’s slope between thymoma and mediastinal lymphoma, indicating a potential for distinguishing lesions with different etiology based on this spectral characteristic.^[19]^ Previous studies have also demonstrated that the slope of the spectral curve could be used in distinguishing malignant from benign SPNs.^[13,20,21]^

In our study, we observe that malignant lesions exhibit significantly higher slope of HU compared to benign lesions following contrast administration. This difference is particularly evident in images acquired at lower keV monochromatic images, as the attenuation of iodine is greater at lower energies. Similar findings have been reported in several studies with a comparable cohort, showing AUC values ranging from 0.66 to 0.8.^[13,20,21]^

#### 4.3.3. IC

The basic theory in tumor development suggests that growing tumors acquire blood supply from adjacent tissues. This is facilitated by increased permeability of tumor capillaries, resulting in high blood flow and distribution in the intravascular and extracellular spaces. As a result, malignant tumors exhibit rapid and strong contrast enhancement compared to benign lesions. Malignant lesions tend to develop more blood vessels to support their rapid growth, leading to stronger enhancement. This phenomenon is well-documented in previous studies.^[13,22,23]^

DECT allows the generation of IC maps, which are considered useful for assessing tumor vascularity. Our study’s findings regarding IC are consistent with previous research, which has reported AUC, sensitivity, and specificity values in the ranges of 0.75 to 0.82, 51% to 96%, and 63% to 92%, respectively. Li et al and Zegadlo et al focused on the diagnostic value of kVp-switching dual-energy CT in necrotic lung lesions and SPNs. They conducted scans on 50 benign SPNs and 86 malignant SPNs, demonstrating higher IC values in malignant lesions compared to benign lesions.^[16,21]^

#### 4.3.4. Normalized iodine concentration

The IC values of lung lesions are normalized to those of the thoracic aorta to derive the NIC. This approach offers advantages. It helps minimize variations caused by the patient’s circulation status and scanning times, making the comparison of different cases more reasonable and reliable.^[13,20]^

Due to the complex pulmonary vascular anatomy of lung lesions, which receive dual blood supply from the branches of aorta and pulmonary artery, and drain into the pulmonary vein, assessing lesion enhancement in the lungs is challenging compared to lesions with a single blood supply. Therefore, selecting the standard phase for evaluating lesions becomes difficult. Fortunately, previous studies have demonstrated that NIC values in the venous phase surpassed the arterial phase in differentiating between malignant and benign nodules.^[13,16,20]^ Thus, in our study, we normalize the IC values of lung lesions to the thoracic aorta, pulmonary artery, and pulmonary vein in the venous phase. However, even though the NICs had significant difference between benign lesion and malignant lesion under Mann–Whitney *U* test (*P* value between 0.001 and 0.003) in our study, the AUC value of NICa in our study is inferior to that of Zhang et al and Wen et al study (AUC = 0.93–0.96).^[13,20]^

It may have 2 reason. First, even though the NICs have significant difference between benign lesion and malignant lesion with Mann–Whitney *U* test (*P* value between 0.001 and 0.003) in our study, our preliminary study (N = 71) from small number patients supplied limited sensitivity and specificity as compared with Wen et al study^[13]^ (N = 135). In the future, we will recruit a larger number of database to improve it. Second, in the studies by Zhang et al and Wen et al,^[13,20]^ they excluded cases of acute inflammation during collecting the benign cases. However, in our study, we still included cases of acute inflammation or infection, such as fungal or cryptococcal infection, pulmonary abscess and septic embolism because they could appear nodular like lesions. Distinguishing them from malignancies are crucial and we also want to know if they possess different characteristics on spectrum CT comparing with malignancies. Actually, some studies found lesions of acute inflammation have high vascularity and vascular permeability, which are similar as malignancy.^[24,25]^ The relative low AUC(0.70–0.79) of our second part study comparing with other studies was probably due to this effect.

### 4.4. Limitation

Some limitations may be considered for this study. First of all is the relative small case capacity, especially benign lesions, due to single center study. In future investigations, we aim to collect a larger case capacity to reduce the effects of this limitation.

Secondly, other studies have indicated that nodules with acute inflammation may have dilated capillaries stimulated by inflammatory factors, leading to higher values of spectral parameters similar to malignant lesions.^[24,25]^ This might potentially interfere with our study results. Third, we did not observe the intra- or interobserver variance to quantify the reliability and reproducibility of the measurements.

## 5. Conclusion

In our study, malignant and benign pulmonary lesions demonstrated significant difference on multiple spectrum CT derived parameters, allowing promising capability of distinguishing malignant from benign lesions on contrast-enhanced dual-energy spectral CT, avoiding the unnecessary invasive procedure or surgery.

## Acknowledgments

Jun-Peng Chen, Department of Medical Research, Taichung Veterans General Hospital for biotechnology consultation.

## Author contributions

**Conceptualization:** Tse-Pang Ko, Yu-Pin Chang.

**Data curation:** Tse-Pang Ko.

**Formal analysis:** Tse-Pang Ko.

**Investigation:** Tse-Pang Ko.

**Methodology:** Tse-Pang Ko, Yu-Pin Chang.

**Project administration:** Tse-Pang Ko, Yu-Pin Chang.

**Resources:** Tse-Pang Ko.

**Software:** Tse-Pang Ko.

**Supervision:** Yu-Pin Chang, Jyh-Wen Chai.

**Validation:** Tse-Pang Ko.

**Visualization:** Tse-Pang Ko.

**Writing – original draft:** Tse-Pang Ko.

**Writing – review & editing:** Yu-Pin Chang.
